# What Hispanic parents do to encourage and discourage 3-5 year old children to be active: a qualitative study using nominal group technique

**DOI:** 10.1186/1479-5868-10-93

**Published:** 2013-08-06

**Authors:** Teresia M O’Connor, Ester Cerin, Sheryl O Hughes, Jessica Robles, Deborah Thompson, Tom Baranowski, Rebecca E Lee, Theresa Nicklas, Richard M Shewchuk

**Affiliations:** 1 Department of Pediatrics, USDA/ARS Children’s Nutrition Research Center, Baylor College of Medicine, Houston, TX, USA; 2 Department of Pediatrics, Academic General Pediatrics, Baylor College of Medicine, Houston, TX, USA; 3 Institute of Human Performance, The University of Hong Kong, Pokfulam Road, Hong Kong SAR, Hong Kong; 4 School of Exercise and Nutrition Sciences, Deakin University, Melbourne, Australia; 5 Department of Health & Human Performance, Texas Obesity Research Center, University of Houston, Houston, TX, USA; 6 Department of Health Services Administration, University of Alabama at Birmingham, Birmingham, AL, USA

**Keywords:** Physical activity, Parenting practices, Hispanic, Preschool child, Qualitative research, Nominal group technique

## Abstract

**Purpose:**

Hispanic preschoolers are less active than their non-Hispanic peers. As part of a feasibility study to assess environmental and parenting influences on preschooler physical activity (PA) (*Niños Activos*), the aim of this study was to identify what parents do to encourage or discourage PA among Hispanic 3-5 year old children to inform the development of a new PA parenting practice instrument and future interventions to increase PA among Hispanic youth.

**Methods:**

Nominal Group Technique (NGT), a structured multi-step group procedure, was used to elicit and prioritize responses from 10 groups of Hispanic parents regarding what parents do to encourage (5 groups) or discourage (5 groups) preschool aged children to be active. Five groups consisted of parents with low education (less than high school) and 5 with high education (high school or greater) distributed between the two NGT questions.

**Results:**

Ten NGT groups (n = 74, range 4-11/group) generated 20-46 and 42-69 responses/group for practices that encourage or discourage PA respectively. Eight to 18 responses/group were elected as the most likely to encourage or discourage PA. Parental engagement in child activities, modeling PA, and feeding the child well were identified as parenting practices that encourage child PA. Allowing TV and videogame use, psychological control, physical or emotional abuse, and lack of parental engagement emerged as parenting practices that discourage children from being active. There were few differences in the pattern of responses by education level.

**Conclusions:**

Parents identified ways they encourage and discourage 3-5 year-olds from PA, suggesting both are important targets for interventions. These will inform the development of a new PA parenting practice scale to be further evaluated. Further research should explore the role parents play in discouraging child PA, especially in using psychological control or submitting children to abuse, which were new findings in this study.

## Background

Physical activity (PA) has many health benefits, and has been recommended for a healthy lifestyle and to maintain healthy weight [1]. According to the National Association of Sports and Physical Education, preschool children should get at least 60 minutes of structured PA and at least 60 minutes of unstructured PA daily through play [2]. However, in one small study, Hispanic preschool children only spent 3.4% of their day engaged in moderate-to-vigorous physical activity [3]. Hispanic children may also be less active than non-Hispanic white children [4]. Hispanic children represent the fastest growing ethnic minority in the US [5], and are disproportionately affected by overweight and obesity, even among 2-5 year olds [6]. To address the current problem of overweight and obesity in Hispanic children, it is important to identify modifiable factors associated with PA in early childhood.

Parents impact their children’s socialization and behaviors [7] and parenting has received increasing attention as a target to promote healthy lifestyle behaviors among children. A systematic review identified parents as important influences on children’s PA for elementary school aged children, through active modeling, direct involvement, encouragement, and providing transportation [8]; while a second review of parent influences concluded that only parental support (informational, emotional, appraisal, and instrumental) was consistently associated with children’s PA [9]. A meta-analysis found a moderate positive association for parental support and modeling PA behaviors with child and adolescent PA [10]. Beets et al. proposed that parents provide both tangible (instrumental and conditional) and intangible (motivational and informational) support for their child to be active, which have been related to children’s PA [11]. However, studies have focused on what parents do that may positively influence children’s PA behaviors, with less emphasis on what parents do that may negatively influence, or discourage children’s PA.

Parents may have greater influence on elementary school aged children’s, than adolescents’ , PA [8]. This is likely due to developmental differences, and suggests that parents are especially important in influencing children’s PA at even younger ages. However, few studies have assessed what parenting practices are used by parents with 3-5 year-old children or ethnic minority parents to encourage or discourage their PA. The systematic review by Trost et al [9] showed inconclusive results of the impact of parental support on preschoolers’ PA due to limited number of studies and also recommended an urgent need to assess the influence of parents on ethnic minority children’s PA. To our knowledge, no multi-dimensional PA parenting practice scales exist for parents with preschool aged children, nor are any specific for Hispanic children. Cultural variables influence children’s PA behaviors [12] and parenting [13], so PA parenting practice instruments developed for Hispanic parents should be informed by them directly.

As the initial part of a feasibility study to assess environmental and parenting influences on their preschool-aged child’s PA (*Niños Activos*), this study aimed to identify parenting practices used by Hispanic parents of 3-5 year-old children that may encourage or discourage PA in their child. Identification of important and salient parenting practices can help inform the development and validation of a comprehensive instrument for PA parenting practices for Hispanic families with preschool aged children, and may provide targets for change in future interventions.

## Methods

A structured qualitative study was conducted with parents of 3-5 year-old Hispanic children to generate lists of parenting practices parents report may positively or negatively impact their preschool aged child’s PA. A convenience sample of parents was recruited via fliers posted or distributed at local community centers, churches, health fairs, food fairs, and retail outlets in Houston, Texas, as well as notices posted on the Baylor College of Medicine and USDA/ARS Children’s Nutrition Research Center volunteer web-sites, and calls to research volunteers listed on the CNRC research volunteer database. Parents were included if they self-identified as Hispanic and reported being the primary caregiver/parent of at least one Hispanic 3-5 year-old child living in the Houston area. Exclusion criteria were a parent or primary caregiver of a child with a disease (e.g., physical disability, severe asthma) or cognitive disability (e.g., William’s and Down’s syndromes) preventing them from participating in regular PA; parent who did not self-identify as Hispanic or was unable to read and write in English or Spanish. Parents were screened in person or by phone, provided informed written consent prior to participating in the qualitative study, and completed a demographic survey. Research staff members were bilingual, and participants were given the choice to participate in sessions conducted in English or Spanish. The Baylor College of Medicine Institutional Review Board approved all study materials and protocols.

Nominal Group Technique (NGT) [14,15] is a structured multi-step group procedure that can elicit and prioritize responses from a group of people in reaction to a question or problem. This technique has been used in a number of different contexts to generate ideas or allow a group to come to consensus [16-18]. NGT has advantages over traditional focus groups and other qualitative research methods to address the research aim, for several reasons [19,20]. In order to help inform the inclusion of items in a PA parenting practice instrument, parent input was needed that was both qualitative (idea generating) and quantitative (item ranking). NGT offers an equitable way for this to occur and engages all participants in a group setting to generate lists of ideas [14,15,19]. Due to its structured method, one participant in the group cannot dominate the discussion and constrains the group leader to have less influence (and therefor less potential bias), as may occur in traditional focus groups [19]. NGT has been used as an efficient method for obtaining participant input in order to develop new items for a parenting feeding scale [21].

Ten groups of Hispanic parents participated in NGT procedures to identify what parents do to encourage (5 groups) or discourage (5 groups) preschool children to be active. Staff were trained in NGT methodology by a NGT expert and co-author (RMS) during a two-day training session that included didactic training and practice sessions with feedback. At least two staff facilitated each session. It is possible there are differences in parenting practices by socio-economic status; therefore parental education was used as a proxy for SES. Five groups consisted of Hispanic parents with lower education (less than high school) and 5 with higher education (high school or greater). Each group was given an overview of the NGT procedures and instructions and then asked one of two questions: “What are things that parents do that encourage 3-5 year old children to be active?” (3 of the 5 groups reported higher education) or “What are things that parents do that discourage 3-5 year-old children to be active?” (2 of the 5 groups reported higher education). The terminology and questions used in the groups were vetted by Hispanic parents in a series of cognitive interviews (n = 12) in English or Spanish conducted prior to the NGT sessions. After the question was posed, participants were asked to individually and independently generate as many different, concise responses as possible. During this process, participants were prompted by the statement: “We are interested to learn about things parents do on purpose or not on purpose that encourages (or discourages) 3-5 year old children to be active.” Once responses had been generated, the lead facilitator used a round-robin approach to allow each participant to nominate a new response to the question from their list. This continued until all participants had the chance to exhaust their ideas.

The lead facilitator reviewed each nominated item with the group, allowed the participants to clarify any ambiguous responses and gave participants an opportunity to combine equivalent responses. Once the group’s list of responses was finalized, participants were asked to confidentially prioritize the “three things that parents do that you think are the most likely to encourage (or discourage) their 3-5 year old child to be active,” from the full list of responses and then rank the three items from 1-3. The response each participant perceived as most likely was assigned three votes (the most votes) by that participant, the second most likely two votes, and third most likely, one vote. The facilitator then collected and tabulated the votes for each response and presented a list of the responses that received the most votes to each group. All the responses (items) generated by each group were entered into a database, along with the votes assigned to each response and their rank order. The responses from the five groups that addressed each question were aggregated and items that were identical or very similar were combined by several of the authors based on similarities in parent behaviors (such as responses related to TV limits). Scores for the same or very similar items were combined across groups. The authors reviewed these aggregations, differences in opinions were discussed and consensus agreement was determined. This allowed all the responses to be ranked by number of votes and number of groups that endorsed it as most likely to encourage (or discourage) Hispanic preschool children’s PA across the five groups that addressed the same question.

## Results

Seventy four Hispanic parents or legal guardians of 3-5 year-old children participated in ten NGT sessions (n = 4-11/group) between September-December 2010 (Table 1). The majority of participants were women (92%), preferred to participate in a Spanish speaking group (82%), and reported a family income of under $20,000/year (55%). About half reported having a female preschool child (53%), and 45% reported having at least a high school diploma. Eight of the group sessions were conducted in Spanish (split evenly by the questions posed). Three of the five groups that addressed ways parents encourage their preschool child to be active were attended by parents who had at least a high school diploma, compared to two of the five groups that addressed ways parents discourage their preschool child to be active. The 10 NGT groups generated 20-46 and 42-69 responses/group, respectively for practices that encourage or discourage PA. Eight to 18 responses/group were elected as the most likely to encourage or discourage PA (Table 2).

**Table 1:** Participant characteristics (n = 74)

**Family characteristic**	**n (%)**
Language (Spanish)	61 (82%)
Parent Gender (female)	68 (92%)
Child gender (female)	39 (53%)
Family Income	
<$20,000/year	41 (55%)
$20,000- < $50,000/year	30 (41%)
≥ $50,000/year	3 (4%)
Parent Education	
< High School	41 (55%)
≥ High School	33 (45%)

**Table 2:** Group characteristics

	**Parent practices that *Encourage ***	**Parent practices that *Discourage ***
	**physical activity**	**physical activity**
Number of groups that addressed question	5	5
Number of groups conducted in Spanish	4	4
Number of groups reporting High Education ^a^	3	2
Number of participant/group, mean (range)	6.2 (4-8)	8.6 (7-11)
Items generated/group, mean (sd)	35.2 (9.6)	44.8 (9.7)
Items elected “most likely”/group, mean (sd)	11 (2.5)	14.8 (2.8)

^**a**^High Education- at least a high school degree.

### Parenting practices that encourage child physical activity

‘Feeding the child well’ , ‘signing child up for sports’ , ‘participating in activities with the child’ , ‘dancing with them’ , ‘playing sports with the child’ , and ‘letting the child see parent be active’ , emerged as the responses with the greatest number of votes across the five groups that discussed ways to encourage PA among preschool children. Although ‘taking the child to the park’ did not receive as many votes as these practices, it was elected as most likely by the greatest number of groups (four of the five groups). Four of the encouraging parenting practices were identified as most likely by three of the five groups: ‘signing child up for sports’ , ‘dancing with them’ , ‘letting the child see parent be active’ , ‘playing with child’. All the other practices listed were identified as likely to encourage PA of Hispanic 3-5 year-old children by one or two of the groups (Table 3). ‘Feeding the child well’ , ‘giving them activities appropriate for their age’ , and ‘focusing on what our children like the most’ were only endorsed by the two groups with lower education, and ‘cutting TV time’ was only endorsed by two of the three higher education groups.

**Table 3:** **Aggregated results across 5 groups for parenting practices most likely to *ENCOURAGE *physical activity**

**Responses**	**Total votes**	**Number of groups**	**Education**
	**(across groups)**	**endorsed response**	**level of group**
Eating well and sleeping well; Feeding them well.	23	2	Low, Low
Signing them up in sports.	22	3	Low, High, High
Participating in the children’s activities; Doing activities with them.	15	2	Low, High
Teaching them to dance and sing; Dancing with them; Playing music to encourage them to dance and act; Playing children’s music with steps to follow.	14	3	Low, High, High
Playing a sport as a family; Playing with the children their favorite sport; Playing a sport with them.	12	2	Low, High
If the parents exercise then so will the children; Parent’s should set an example; Letting them see you be active.	11	3	Low, High, High
Playing (or playing ball) with them.	8	3	Low, Low, High
Going with them to the park; Taking them to the park; Playing at the park; Taking them to the park	7	4	Low, High, High, High,
Don’t watch too much television; Cutting TV time.	7	2	High, High
Not being sick.	6	1	Low
Jumping on (Use) trampolines.	5	2	Low, High,
Going out to walk; Taking them out of the house to the park to walk.	5	2	Low, High,
Inviting them to go outside; Letting them go outside to play.	5	2	Low, High
Looking for age appropriate games that helps them to be agile; Giving them activities appropriate for their age	5	2	Low, Low
Focusing on what our children like the most	4	1	Low, Low
Riding a bicycle; Letting them ride bicycles safely.	3	2	Low, High
Motivating the children.	3	1	Low
Letting them run.	3	1	Low
Getting active video games.	3	1	High
Going camping.	3	1	High
Teaching them different ways to exercise.	3	1	High
Jumping rope.	2	1	High
Being active (children) during their school activities.	2	1	Low
Giving them different options.	2	1	Low
Teaching them that being active is good for their health.	2	1	Low
Helping with outside chores such as gardening.	2	1	High
Reading with them.	2	1	High
Parents should be more creative.	2	1	High
Taking them to a picnic.	1	1	Low
Having designated play time.	1	1	High
Having outside toys available for them.	1	1	High
Visiting family members.	1	1	High
Imitating animal movements.	1	1	High

Low education- not completed high school or equivalent; High education- completed high school degree or higher.

### Parenting practices that discourage child physical activity

‘Allowing child to watch a lot of TV’ , ‘verbal criticisms and insults’ , ‘emotional or physical abuse’ , ‘allowing them to play a lot videogames’ , ‘not letting them be children’ , and ‘lack of time to engage with child’ , emerged as the responses with the greatest number of votes across the five groups that addressed things parents do that discouraged children from being active. The practices with greatest consensus across groups were ‘allowing child to watch a lot of TV’ and ‘allowing them to play a lot videogames’ , (identified by four of the five groups); and ‘verbal criticisms and insults’ , ‘not letting them be children’ , ‘lack of time to engage with child’ , ‘not giving them a healthy diet’ , and ‘preventing them from being active because of fear they would get hurt’ (identified by three of the five groups) (Table 3). ‘Not taking them to the park’ , and ‘not motivating them to be active at an early age’ were only endorsed by two of the three lower educated groups, while ‘babying them’ was only endorsed by the two higher educated groups.

## Discussion

While the existing literature on PA parenting practices has primarily focused on practices intended to positively impact mostly older children’s PA [8,10,11], this study identified parenting practices that either encourage or discourage PA, specifically among Hispanic preschool children. A systematic review of correlates of preschool children’s PA identified only five studies that investigated parenting variables associated with preschool aged children’s PA [22]. Most of these studies assessed PA parenting practices via research staff observational coding using similar procedures in relatively small samples [23-26] or had parents report on their own PA as a proxy for parental modeling of PA [27]. These previous studies found positive associations of parent PA and family interactions with children’s observed PA [22]. However, the three studies that assessed parental verbal prompts to encourage or discourage PA with observations in the home, found no associations with children’s PA; while indoor and outdoor play rules were negatively associated with children’s PA [22]. The present study suggests that Hispanic parents with 3-5 year-old children use multiple ways, other than just verbal prompts, to encourage or discourage their child to be physically active.

The parenting practices that received the strongest endorsements in this study to encourage PA among Hispanic preschoolers included various ways in which parents engage with their child in active play, such as interacting, playing, dancing, or playing sports. This suggests active engagement may be an important PA parenting practice influence among 3-5 year old children. Similar to previous studies [22] parental modeling of PA was also frequently endorsed as a way parents encourage their child to be active. Some groups identified that children need to see their parents be active, suggesting this may be different than just having an active parent. Signing one’s child up for sports and taking them to the park were also endorsed across several groups and is similar to parental logistic support previously reported as influential for older children’s PA [11,28]. Several of the encouraging items are similar to items from the Family Influence Scale used among older children [29]. Participants in this qualitative study also associated feeding children well with encouraging them to be active. Parents appeared to appreciate the interactive effects of diet and PA in promoting children’s health, which has been noted in other qualitative studies focused on nutrition with Hispanic mothers of 3-5 year-old children [30].

Hispanic parents of preschool aged children endorsed many ways that parents discourage their children from being active. Promoting screen media use (both TV viewing and playing electronic games), which are often sedentary behaviors, were identified by the majority of the groups as most likely to discourage children’s PA. Previous work with Hispanic parents of 5-8 year-old children informed by focus groups similarly identified restricting screen media as a primary way Hispanic parents try to encourage their child to be more active [31]. Multiple groups in the present study also endorsed that parental criticism, intimidation, or insults of a child would discourage a child from being active. These forms of parenting behaviors are commonly thought of as psychological control in developmental psychology and have consistently been associated with less desirable outcomes in youth [32]. To our knowledge the effect of parental psychological control on children’s structured PA (such as sports participation) or unstructured PA (such as play) have not been explored and warrant further study. Two of the four groups suggested that children who are emotionally or physical abused by their parents will be discouraged from being more physically active. This has also not previously been identified in the PA literature. There is some evidence that abused children play differently from non-abused children [33,34], showing more delayed cognitive and physical play skills and decreased playfulness [33]. This may translate into less PA since much of preschool children’s PA is obtained through unstructured play, but to our knowledge there is no data to support these hypothesized associations; thus, further investigation is needed to more fully examine this. The groups that generated ideas for ways that parents discourage PA identified more responses per group than those that discussed practices that encourage child PA. However, those groups were also slightly larger such that the average number of items generated per participant was similar between the two types of groups.

There were only a few differences in the ideas generated for how parents encourage or discourage children to be active, between the high and low education groups (Tables 3, 4). Some of these differences may relate to income differences in the families. For example, families with lower education may represent a lower socio-economic group that has less availability and accessibility of parks or food, explaining why they were more likely to mention ‘Not taking them to the park’ , or ‘Feeding the child well’ as ways to discourage or encourage child PA respectively. The significance of these differences should be further explored in larger quantitative studies.

**Table 4:** **Aggregated results across 5 groups for parenting practices most likely to *DISCOURAGE *physical activity**

**Responses**	**Total votes**	**Number of groups**	**Education**
	**(across groups)**	**endorsed response**	**level of group**
Allowing them to watch TV for long periods of time; Putting the television on all day; Letting them watch a lot of television; Letting them watch a lot of television.	21	4	Low, Low, High, High
Telling them they are not in proper condition to play a sport; Criticizing them all the time; When they are insulted by their parents; Intimidate them; Telling them negative words.	20	3	Low, Low, High
Abusing them emotionally; When children are abused by their parents or other family member; Physical abuse.	17	2	Low, High
Giving kids electronics to entertain them; Buying them a lot of video games; Purchasing electronics specifically for them; Children playing with electronic games; Allowing them to play a lot with video games.	17	4	Low, Low, Low, High
Not letting them do what they want; Not letting them be children; Giving them responsibilities not adequate for their age.	14	3	Low, Low, High
Busy; When parents work too much; When there is no time to play with them.	13	3	Low, High, High
Not giving them a healthy diet causing them to be obese; Not giving them a healthy diet; Changing their diet so they won’t have energy, Letting them be overweight.	12	3	Low, Low, High,
Not instilling in them exercise; Not teaching them the importance of physical activity	11	1	Low
Not paying attention to them.	11	2	Low, High
By not being examples ourselves; Not giving them an example.	10	2	Low, High
Not interacting or play (sharing) games with them; Parents not participating in their children’s activities.	9	2	Low, High
Not registering them in classes that involve physical activity because of lack of money.	7	1	Low
Not having the time to take them to the park; Not taking them to the park.	7	2	Low, Low
Not motivating them to be active at an early age; Not motivating the children; Not motivating them in sports.	7	2	Low, Low
Scolding them for any mischief; Punishing them.	7	2	Low, High
Keeping your kids indoor all day; Keeping them at home at all times.	6	2	Low, High,
Not providing different indoor activities; Not educating them on different physical activities.	5	1	High
Babying them; Spoiling them.	5	2	High, High
Instilling fear and insecurity so they won’t run and jump from high places; Prevent them from getting hurt; Being scared about them hurting themselves.	5	3	Low, Low, High
Domestic Violence.	5	1	Low
As a result of an illness/medical problem.	5		High
Keeping them occupied with quiet games; Play table games.	4	1	Low
Forcing children to participate in their parent’s activities.	4	1	High
Don’t let them play outside for safety reasons.	3	1	High
Spending time with them.	3	1	Low
Lack of recreation.	3	1	Low
Taking them in the stroller when the child is too big.	3	1	Low
Rewarding them for being still; Reward them for behaving well.	3	1	Low
Not doing many outdoor activities.	2	1	High
Reading to them.	2	1	Low
Driving them every where like to school.	2	1	Low
Parents not exercising	2	1	Low
Not giving them love and understanding.	2	1	Low
Not participating in chores.	2	1	Low
Not letting them socialize with other children.	2	1	High
Taking away their favorite toy.	2	1	High
Sending them to sleep early.	2	1	High
Teaching them to be patient when they want something.	1	1	Low
Not going out to walk with them.	1	1	Low

Low education- not completed high school or equivalent; High education- completed high school degree or higher.

NGT is formal structured group process for idea generating [14] that promotes more even rates of participation across participants [15] and is therefore a useful method to generate potential items for new psychosocial or behavioral scales. However, a limitation in using NGT procedures is not allowing for elaborations of the reasons why parents nominated or elected items as most likely to encourage or discourage Hispanic preschool aged children to be active. Thus, there was no opportunity to explore what parents thought would happen to a child’s PA behaviors if parents used psychological control or were physically or emotionally abusive, nor how common these behaviors may be. Both of these emerging themes warrant further study to see how important they may be in influencing children’s PA while the child is young as well as when they get older. Future studies should also explore how environmental variables [35,36], personal characteristics, as well as parents’ perceptions of their child PA competence [37] and the neighborhood environment [38] influence Hispanic parents use of different PA parenting practices. In addition, the structured focus groups were conducted with a convenience sample of Hispanic parents from one US city. The findings from this study may not generalize to other Hispanic parents with preschool aged children or to other ethnic groups.

## Conclusions

Parents generated candidate PA parenting practices that they believed encourage or discourage PA among preschoolers. The parenting practices identified in this study will inform the development of a new PA parenting practice scale which will be further evaluated for its psychometric properties and reliability. Future studies will also need to assess the new PA parenting practice scale’s predictive validity for preschool children’s PA. Qualitative studies, such as focus groups or semi-structured interviews, should further investigate parents’ beliefs that psychological control and physical or emotional abuse would affect a child’s PA.

## Abbreviations

PA: Physical activity; NGT: Nominal group technique.

## Competing interests

We the authors, have no financial or non-financial competing interests with the contents of this manuscript.

## Authors’ contributions

TO was the PI on the NIH funded R21 grant, oversaw all aspects of the staff training and study protocols, analyzed the data, and drafted the manuscript. EC initially developed the Ninos Activos study design, wrote the grant, informed the development of the group questions, and critically read and approved the manuscript. JR was the study coordinator on the project, informed the development of the group questions, and facilitated the group sessions with other study staff. SH is a parenting expert in child feeding with previous experience using NGT methods who informed the development of the group questions, assisted with data analyses, and critically read and approved the manuscript. DT is a qualitative researcher who trained the study staff in cognitive interview methodology to vet the NGT questions, informed the development of the group questions, and critically read and approved the manuscript. TB provided input on the Ninos Activos study and critically read and approved the manuscript. RL provided input on the Ninos Activos study and critically read and approved the manuscript. TN provided input on the Ninos Activos study and critically read and approved the manuscript. RS is an expert in Nominal Group Technique methodology and helped inform the study protocol, trained the staff who conducted the group sessions, helped analyze the data, and critically read and approved the manuscript. All authors read and approved the final manuscript.
